# 射频消融在EGFR-TKIs治疗非小细胞肺癌后局部进展的初步临床应用

**DOI:** 10.3779/j.issn.1009-3419.2016.12.09

**Published:** 2016-12-20

**Authors:** 宝东 刘, 元博 李, 牧 胡, 磊 刘, 坤 钱, 若天 王

**Affiliations:** 100053 北京，首都医科大学宣武医院胸外科 Department of Thoracic Surgery, Xuanwu Hospital, Capital Medical University, Beijing 100053, China

**Keywords:** 表皮生长因子受体酪氨酸激酶抑制剂, 肺肿瘤, 局部进展, 射频消融, Epidermal growth factor receptor tyrosine kinase inhibitors, Lung neoplasms, Locally progression, Radiofrequency ablation

## Abstract

**背景与目的:**

表皮生长因子受体酪氨酸激酶抑制剂(epidermal growth factor receptor-tyrosine kinase inhibitors, EGFR-TKIs)是*EGFR*敏感突变非小细胞肺癌患者的主要治疗手段之一，但是部分患者在EGFR-TKIs治疗有效后出现原发灶局部进展。本文研究了射频消融在EGFR-TKIs治疗*EGFR*突变非小细胞肺癌后局部进展的临床应用结果。

**方法:**

入组符合条件的28例非小细胞肺癌患者，肺部肿瘤经过射频消融和后续的EGFR-TKI或化疗，观察其安全性及治疗效果。

**结果:**

所有患者无围手术期死亡。平均随访17.25个月。局部进展率为10.7%(3/28)，局部进展时间平均为16.6个月。平均肿瘤无进展时间为(24.55±5.36)个月(95%CI: 14.04-35.05)，平均总生存时间(overall survival, OS)为(25.57±5.45)个月(95%CI: 14.88-36.27)。射频消融后续治疗分为EGFR-TKIs组和化疗组，两组平均肿瘤无进展时间分别为(27.82±7.58)个月(95%CI: 12.97-42.68)和(17.88±3.76)个月(95%CI: 10.52-25.25)(*P* > 0.05)；平均OS分别为(29.42±7.68)个月(95%CI: 14.36-44.48)和18.44±3.87(95%CI: 14.89-36.27)(*P* > 0.05)。

**结论:**

针对*EGFR*敏感突变的非小细胞肺癌患者，应用EGFR-TKIs治疗有效后出现原发灶局部进展，射频消融可提高局部控制率，并延长肿瘤无进展生存期和总生存期。

## Abstract (en)

**Background and objective:**

Epidermal growth factor receptor tyrosine kinase inhibitors (EGFR-TKIs) is one of the foundamental treatment for non-small cell lung cancer (NSCLC) with *EGFR* mutation, however some patients might develop locally progression in primary site. The aim of this study is to assess the clinical application of radiofrequency ablation after locally progression of NSCLC while receving EGFR-TKIs.

**Methods:**

Twenty-eight eligible NSCLC patients were enrolled. Efficacy and Safety data of radiofrequency ablation followed by EGFR-TKIs or chemotherapy were collected.

**Results:**

None of patients had died during peri-operation period. The average follow-up time was 17.25 months. Locally progression rate was 10.7% (3/28), and locally progression time was 16.6 months. The average progression-free survival was (24.55±5.36) (95%CI:14.04-35.05), and the average overall survival was (25.57±5.45)(95%CI:14.88-36.27). Patients were divided into EGFR-TKIs group and chemotherapy group after radiofrequency ablation. The average progression-free survival of the two groups were (27.82±7.58)(95%CI:12.97-42.68) and (17.88±3.76)(95%CI:10.52-25.25)(*P* > 0.05) respectively. The average OS (overall survival) was (29.42±7.68)(95%CI:14.36-44.48) and (18.44±3.87)(95% CI:14.89-36.27)(*P* > 0.05) in two groups.

**Conclusions:**

Radiofrequency ablation combined with EGFR-TKIs or chemotherapy could prolong progression-free survival and overall survival of *EGFR* mutant NSCLC patients who had developed locally progression in primary site during EGFR-TKIs treatment.

表皮生长因子受体(epidermal growth factor receptor, *EGFR*)敏感突变的非小细胞肺癌(non-small cell lung cancer, NSCLC)患者能够从EGFR酪氨酸激酶抑制剂(EGFR -tyrosine kinase inhibitors, EGFR-TKIs)的治疗中获益。然而经过一段时间的EGFR-TKIs治疗后，几乎所有患者都会出现耐药，耐药主要表现为三种模式，即快速进展、缓慢进展和局部进展^[1]^。本研究回顾性分析了接受EGFR-TKIs治疗的*EGFR*突变NSCLC肺原发灶局部进展后射频消融(radiofrequency ablation, RFA)的治疗效果。

## 1 材料与方法

### 1.1 临床资料

入组标准：①*EGFR*敏感突变；②EGFR-TKIs初治一线治疗(即未接受针对肿瘤的治疗，如放化疗)有效，但随后出现耐药，肺原发灶出现局部进展的非小细胞肺癌(non-small cell lung cancer, NSCLC)患者；③高龄、心肺功能差、拒绝手术或放疗的早中期NSCLC患者；④或者存在远处转移的晚期NSCLC患者无法接受手术切除或放疗。接受EGFR-TKIs治疗后4周复查胸部计算机断层扫描(computed tomography, CT)，以后每8周复查一次，肺原发灶局部进展时采用射频消融。

### 1.2 射频消融

术前检查包括血常规、肝肾功能、肿瘤标志物、胸部CT、肿瘤SPECT或正电子发射型计算机断层显像(positron emission computed tomography, PET)、腹部B超、骨扫描、头颅核磁。经患者本人和家属签署知情同意书。采用“四步穿刺进针法”^[2]^：①注射器皮肤定位：穿刺点用1%-2%利多卡因局部麻醉，充分麻醉至胸膜，留置5 mL注射器。再次CT扫描，在CT图像上观察注射器针头与肿瘤及其周围组织结构的位置关系，再次确认体表穿刺深度与角度。②射频针肺内定位：根据肿瘤的大小，选择相应规格的RFA针(美国RITA公司生产的射频针)，避开肋骨、大血管、肺大泡，将射频针按事先测得的方向和角度快速到达肿瘤附近。再次CT扫描，观察射频针针尖与肿瘤及周围组织结构的关系。③射频针瘤内定位：调整射频针穿刺角度，根据测量深度继续进针，将射频针插入瘤体内。④射频针瘤内布针：根据肿瘤的大小、形状及与周围组织结构的关系，微调射频针的穿刺深度和角度(不同的射频针设计不同，因此针尖的位置要求也不同)，理想消融范围要包括肿瘤边缘外0.5 cm-1 cm的组织。消融结束后再次CT扫描，观察肿瘤及周围相关组织结构变化和有无气胸、出血等并发症。确定无异常后，患者卧床送回病房静卧2 h。术后可能出现发热、咯痰带血等症状，给予预防性抗生素和止血等对症处理。

### 1.3 后续治疗

射频消融后，患者继续口服原EGFR-TKIs或其他分子靶向药物治疗，或者改用化疗。

### 1.4 随访和统计学处理

所有患者在射频消融后4周复查胸部CT，以后每3个月复查一次，根据改良实体肿瘤疗效评价标准(Response Evaluation Criteria in Solid Tumors, RECIST)判定近期疗效^[3]^，包括完全消融、不完全消融和局部进展，局部进展者再次消融。远期生存观察肿瘤无进展生存期(progression free survival, PFS)和总生存(overall survival, OS)。

### 1.5 统计学方法

采用SPSS 22.0软件包处理，生存分析采用*Kaplan-Meier*分析和*Log-rank*检验。以*P* < 0.05为差异有统计学意义。

## 2 结果

2009年6月-2016年6月首都医科大学宣武医院胸外科收治的28例NSCLC患者入组。男性患者15例，女性患者13例，年龄35岁-91岁，中位年龄66.5岁。右肺20例，左肺8例。Ⅰ期1例，Ⅱ期1例，Ⅲa期1例，Ⅲb期3例，Ⅳ期22例。病理学检查包括气管镜4例，经胸穿刺活检(transthoracic needle aspiration, TTNA)16例，开胸探查1例，胸腔镜胸膜活检4例，胸水检查3例，组织学类型中腺癌27例，鳞癌1例。所有患者采用扩增阻滞突变系统(amp1ification refractory mutation system, ARMS)法检测标本EGFR基因突变，包括19外显子缺失突变15例，21外显子L858R点突变13例。14例患者接受吉非替尼治疗(250 mg，每日1次)，6例患者接受厄洛替尼治疗(150 mg，每日1次)，8例接受埃克替尼治疗(125 mg，每日3次)。

EGFR-TKIs初治一线治疗的28例NSCLC患者均表现为有效，但随后出现耐药，表现为肺原发灶局部进展，平均肿瘤无进展时间为(13.18±6.52)个月。局部进展的肺部肿瘤接受射频消融，肿瘤大小(4.08±1.79)cm(2 cm-10.5 cm)，每例患者均为1个原发灶，33次首次消融，消融时间平均(25.83±9.30)min(16 cm-50 cm)。所有患者无围手术期死亡，19例消融过程顺利；3例胸痛；4例消融后气胸，其中1例需要胸腔闭式引流；1例迟发性气胸并胸腔闭式引流；1例穿刺时咯血；1例消融后当日出现心衰，经过纠正后好转出院。随诊截止至2016年7月31日，随访1个月-86个月，平均随访17.25个月。20例患者继续口服原EGFR-TKIs或其他靶向药物治疗，8例改用化疗。随访过程中发现3例患者因肺原发灶局部进展而再次消融，局部进展率为10.7%(3/28)，局部进展时间平均16.6个月。平均PFS为(24.55±5.36)个月(95%CI: 14.04-35.05)，平均OS为(25.57±5.45)个月(95%CI: 14.88-36.27)(图 1)。

**1:**
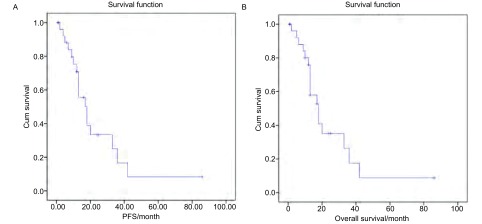
EGFR-TKIs治疗后局部进展患者射频消融的疾病无进展生存期(A)和总生存(B) *Kaplan-Meier* curves of patients in RFA for locally progression after EGFR-TKIs treatment. A: Progression-free survival; B: Overall survival. RFA: radiofrequency ablation; EGFR-TKIs: Epidermal growth factor receptor tyrosine kinase inhibitors.

将射频消融的后续治疗分为EGFR-TKIs组和化疗组，两组平均PFS分别为(27.82±7.58)个月(95%CI: 12.97-42.68)和(17.88±3.76)个月(95%CI: 10.52-25.25)，*Log-rank*(Mantel-*Cox*)检验*P*=0.465。平均OS分别为(29.42±7.68)个月(95%CI: 14.36-44.48)和(18.44±3.87)个月(95%CI: 14.89-36.27)，*Log-rank*(Mantel-*Cox*)检验*P*=0.362(图 2)。

**2:**
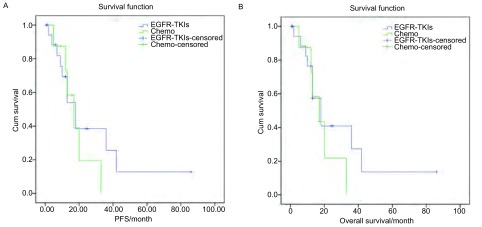
射频消融后续EGFR-TKIs和化疗的患者的疾病无进展生存期(A)和总生存(B) *Kaplan-Meier* curves of patients in different subsequent treatments groups after RFA. A: Progression-free survival; B: Overall survival.

## 3 讨论

以EGFR为靶点的分子靶向药物治疗开启了NSCLC个体化治疗的时代。*EGFR*敏感突变位点通常发生于外显子18-21，包括常见的19外显子缺失(编码E746-A75015个碱基对缺失)和21外显子(L858R)点突变，约占所有*EGFR*突变的85%-90%；少见突变包括18外显子(G719X)点突变、21外显子(L861Q)和20外显子(S7681)，本组全部28例敏感突变患者均属常见突变。目前，国内获批用于临床的EGFR-TKIs包括吉非替尼(Gefitinib，Iressa，易瑞沙)、厄洛替尼(Erlotinib，Tarceva，特罗凯)和国产的埃克替尼(Icotinib，Conmana，凯美纳)。EGFR敏感突变的NSCLC患者能从EGFR-TKIs治疗中获益，多项大型国际多中心随机对照的Ⅲ期临床研究结果均显示EGFR敏感突变者一线治疗有效率为60%-70%，远高于一线化疗的40%^[4-10]^。一线接受EGFR-TKIs治疗的EGFR基因敏感突变的几乎所有NSCLC患者，通常会在治疗后9个月-10个月出现疾病进展，提示出现获得性EGFR-TKIs耐药。EGFR-TKIs获得性耐药的机制可能包括：①T790M、T854A、D761Y、L747S突变；②MET传导通路改变；③生成及上皮-间充质转化(epithelial-mesenehymal transition, EMT)；④其他：PTEN丢失和胰岛素样生长因子受体(insulin-like growth factor receptor, IGFR)激活等。第二代的不可逆EGFR-TKIs阿法替尼(Afatinib)和达克替尼(Dacomitinib)是泛ErbB抑制剂，不但能抑制*EGFR*突变表达，同时还能抑制T790M耐药变异。虽然临床前研究显示成果喜人，不过阿法替尼和达克替尼治疗EGFR-TKIs耐药的临床研究却并不尽如人意。第三代EGFR-TKIs药物有AZD9291、CO-1686和HM61713等。AZD9291是针对T790M研发的第3代EGFR-TKIs，是一种强效口服的不可逆EGFR抑制剂，可抑制EGFR-TKIs敏感和T790M耐药突变，其初步研究结果显示出对T790M突变患者有更好的疗效。一项回顾性研究纳入了227例获得性耐药NSCLC患者，探讨了EGFR-TKIs治疗出现疾病进展后的治疗模式。根据患者的疾病控制时间、肿瘤负荷演变和临床症状6项将患者分为快速进展(疾病控制≥3个月，与以往评估相比，肿瘤负荷快速增加，症状评分达到2)、缓慢进展(疾病控制≥6个月，与以往评估相比，肿瘤负荷轻微增加，症状评分≤1)和局部进展(疾病控制≥3个月，孤立性颅外进展或颅内进展，症状评分≤1)三种临床失败模式。该研究建议针对局部进展的患者建议持续使用EGFR-TKIs加局部治疗^[1]^。

针对肺原发灶局部进展的局部治疗，目前报道较少^[11-17]^，方法包括手术切除^[12, 17]^、肿瘤热消融^[12, 16]^和放疗^[11, 13]^等。尽管有文献报道EGFR-TKIs耐药后可以切除肺原发灶，包括肺叶切除、全肺切除和楔形切除，但是由于口服靶向药物治疗的肺癌患者为高龄、心肺功能差或拒绝手术的早中期NSCLC患者，或者是存在远处转移的晚期NSCLC患者，因此，即使EGFR-TKIs因耐药而出现局部进展，也不可能接受手术切除。局部治疗还可以选择放疗，但是由于同样的理由，部分患者对放疗的耐受性较差，放疗似乎对中心型肺癌可能更好，因为没有呼吸动度的影响，副损伤更小。肿瘤热消融利用热产生的生物学效应直接导致病灶组织中的肿瘤细胞发生不可逆损伤或凝固性坏死，一般在局麻或清醒镇静下进行，消融范围包括靶肿瘤及周围0.5 cm-1 cm的正常组织；具有微创、安全、适形、操作简单、效果可靠、可以重复等优点，是继手术、放疗之后的肿瘤第三大局部治疗手段，目前常用的肺部肿瘤热消融技术有射频消融和微波消融等。

本组病例无围手术期死亡，严重并发症(需要胸腔闭式引流的气胸2例，心衰1例合并少量气胸)发生率为9.1%(3/33)，轻微并发症(胸痛3例、气胸2例、喀血1例)发生率为18.2%(6/33)，与作者以往报道结果类似^[18]^，说明射频消融的操作比较安全。射频消融后的肺部肿瘤局部控制率为89.3%，另外比较EGFR-TKIs和射频消融的PFS也可以看出，射频消融联合后续治疗(EGFR-TKIs或化疗)的局部控制率明显高于单独应用EGFR-TKIs，提示今后临床工作中，只要允许，可以对EGFR敏感突变的患者在口服EGFR-TKIs的同时进行射频消融，即“分子靶向”+“物理靶向”。本文也比较了后续EGFR-TKIs和化疗的PFS和OS，尽管后续EGFR-TKIs治疗组优于化疗组，但是没有统计学差异。由于本研究回顾的病例样本较少，所得数据会有一定偏差，尚无法做出一个肯定的结论。但是针对EGFR敏感突变的NSCLC患者，应用EGFR-TKIs治疗有效后出现原发灶局部进展，射频消融联合EGFR-TKIs或化疗可提高局部控制率，并延长肿瘤PFS和OS。

## References

[1] Yang JJ, Chen HJ, Yan HH (2013). Clinical modes of EGFR tyrosine kinase inhibitor failure and subsequent management in advanced non-small cell lung cancer. Lung Cancer.

[2] Liu BD, Liu L, Hu M (2013). CT-guided percutaneous radiofrequency ablation of pulmonary malignancies located in unusual regions. Jie He Bing Yu Fei Bu Jian Kang Za Zhi.

[3] Liu BD, Zhi XY (2015). Evaluation of local efficacy in pulmonary tumor of Percutaneous radiofrequency ablation. Zhongguo Yi Xue Qian Yan Za Zhi (Dian Zi Ban).

[4] Mok TS, Wu YL, Thongprasert S (2009). Gefitinib or carboplatin-paclitaxel in pulmonary adenocarcinoma. N Engl J Med.

[5] Maemondo M, Inoue A, Kobayashi K (2010). Gefitinib or chemotherapy for non-small-cell lung cancer with mutated EGFR. N Engl J Med.

[6] Mitsudomi T, Morita S, Yatabe Y (2010). Gefitinib versus cisplatin plus docetaxel in patients with non-small-cell lung cancer harbouring mutations of the epidermal growth factor receptor (WJTOG3405): an open label, randomised phase 3 trial. Lancet Oncol.

[7] Han JY, Park K, Kim SW (2012). First-SIGNAL: first-line single-agent iressa versus gemcitabine and cisplatin trial in never-smokers with adenocarcinoma of the lung. J Clin Oncol.

[8] Zhou C, Wu YL, Chen G (2011). Erlotinib versus chemotherapy as first-line treatment for patients with advanced EGFR mutation-positive non-small-cell lung cancer (OPTIMAL, CTONG-0802): a multicentre, open-label, randomised, phase 3 study. Lancet Oncol.

[9] Rosell R, Carcereny E, Gervais R (2012). Erlotinib versus standard chemotherapy as first-line treatment for European patients with advanced EGFR mutation-positive non-small-cell lung cancer (EURTAC): a multicentre, open-label, randomised phase 3 trial. Lancet Oncol.

[10] Shi Y, Zhang L, Liu X (2013). Icotinib versus gefitinib in previously treated advanced non-small-cell lung cancer (ICOGEN): a randomised, double-blind phase 3 non-inferiority trial. Lancet Oncol.

[11] Weickhardt AJ, Scheier B, Burke JM (2012). Local ablative therapy of oligoprogressive disease prolongs disease control by tyrosine kinase inhibitors in oncogene-addicted non-small-cell lung cancer. J Thorac Oncol.

[12] Yu HA, Sima CS, Huang J (2013). Local therapy with continued EGFR tyrosine kinase inhibitor therapy as a treatment strategy in EGFR-mutant advanced lung cancers that have developed acquired resistance to EGFR tyrosine kinase inhibitors. J Thorac Oncol.

[13] Zhang X, Wang B, Lin L (2013). Local treatment combined with EGFR-TKIs for advanced non-small cell lung cancer with solitary progression during EGFR-TKI treatment. Zhongguo Fei Ai Za Zhi.

[14] Kim HJ, Kim WS, Kwon do H (2015). Effects of an epithelial growth factor receptor-tyrosine kinase inhibitor add-on in stereotactic radiosurgery for brain metastases originating from non-small-cell lung cancer. J Korean Neurosurg Soc.

[15] Zhang F (2015). Effective analysis of radiotherapy after locally progression of EGFR-TKIs treatment in advanced non-small cell lung cancers. Zhongguo Shi Yong Yi Yao.

[16] Ni Y, Bi J, Ye X (2016). Local microwave ablation with continued EGFR tyrosine kinase inhibitor as a treatment strategy in advanced non-small cell lung cancers that developed extra-central nervous system oligoprogressive disease during EGFR tyrosine kinase inhibitor treatment: A pilot study. Medicine (Baltimore).

[17] Hishida T, Yoshida J, Aokage K (2016). Long-term outcome of surgical resection for residual or regrown advanced non-small cell lung carcinomas following EGFR-TKI treatment: report of four cases. Gen Thorac Cardiovasc Surg.

[18] Liu BD, Liu L, Hu M (2013). Safety of CT-guided radiofrequency ablation in 400 consecutive patients with unresectable lung neoplasms. Zhongguo Lin Chuang Yi Shi Za Zhi.

